# Paraquat disrupts the blood–brain barrier by increasing IL-6 expression and oxidative stress through the activation of PI3K/AKT signaling pathway

**DOI:** 10.1515/med-2024-1020

**Published:** 2024-09-11

**Authors:** Tao Liu, Fenshuang Zheng, Lin Liu, Hua Zhou, Tao Shen, Yanping Li, Wei Zhang

**Affiliations:** Department of Emergency Medicine, Affiliated Hospital of Yunnan University, Kunming, 650000, Yunnan, China; Department of Emergency Medicine, People’s Hospital of Yuxi City, Yuxi, 653100, Yunnan, China; Department of Emergency Medicine, People’s Hospital of Gejiu City, Gejiu, 661000, Yunnan, China; Department of Emergency Medicine, Affiliated Hospital of Yunnan University, No. 176, Youth Road, Kunming, 650000, Yunnan, China

**Keywords:** paraquat, PI3K/AKT signaling, BBB, IL-6, inflammation.

## Abstract

**Background:**

Paraquat (PQ) is a frequently used herbicide with neurotoxic effects after acute or chronic exposure. Although *in vitro* evidence supports the PQ toxicity to dopamine cells, its *in vivo* effects (especially the chronic exposure) remain ambiguous. In this study, we investigated the effect of chronic PQ exposure on the blood–brain barrier (BBB) damage and the underlying mechanisms.

**Methods:**

Adult male Sprague Dawley rats and primary human brain microvascular endothelial (PHBME) cells were exposed to PQ as the animal and cell models. Evans Blue staining and hematoxylin & eosin staining were conducted to examine the BBB and brain tissue damages. The inflammatory cytokines were quantified via enzyme linked immunosorbent assay. The changes of PI3K/AKT signaling pathway were detected by western blot.

**Results:**

PQ exposure can cause significant pathological lesions in the brain tissues and the BBB. IL-6 and reactive oxygen species levels were found to be significantly upregulated after PQ exposure in both the animal and cell models. PQ treatment could arrest the cell proliferation and migration in PHBME cells. PQ treatment promoted the phosphorylation of PI3K and AKT, and the application of PI3K inhibitor could attenuate PQ-induced IL-6 production, oxidative stress, BBB disruption, and brain tissue damage.

**Conclusion:**

Our study demonstrated that chronic PQ exposure could impair the BBB function and induce brain tissue damage. The overactivation of the PI3K/AKT pathway, consequent upregulation of IL-6 production, and increased oxidative stress appear to mediate the inflammatory damage resulting from PQ exposure.

## 1 Introduction

Paraquat (PQ) is a potent herbicide extensively used in agriculture worldwide due to its unique weed-eradicating properties. However, it has potential toxicity to animals and humans, and its use is prohibited or restricted in the European countries [1,2]. The mortality rate after PQ acute poisoning is >90% since there is no specific antidote or effective treatment [3]. PQ can cause toxicity in multiple organs including lung, liver, kidney, and brain [4]. PQ exposure can cause neurodegeneration which has been recognized as a risk factor for Parkinson’s disease, and the administration of PQ in adult mice suppresses the growth and differentiation of neural stem cells, resulting in decreased nerve activity [5,6]. According to clinical evidence, PQ-induced neurotoxicity and memory dysfunction can be attributed to inflammatory responses [7]. In addition, oxidative stress in many organs due to PQ poisoning is well reported. Intracellular production of reactive oxygen species (ROS) and redox imbalance can lead to tissues damages in the liver and lung [8,9]. In human brain microvascular endothelial cells, PQ treatment was reported to cause the dysregulation of cholesterol biosynthesis proteins [10].

*In vitro* and *in vivo* toxicology studies show that the activities of dopaminergic neurons in specific area of the brain decreases after PQ-induced damages to the substantia nigra, hippocampus, and frontal cortex [11]. There is evidence that PQ can promote neurological disorders by slowly crossing the blood–brain barrier (BBB) via intermediate amino acid transporters and reaching neurotoxic levels [12]. The accumulation of PQ and its derivatives are ineffectively eliminated in the brain compared to other organs such as liver, indicating long-term impact of PQ on the brain [10]. PQ accumulation can culminate in apoptosis and mitochondrial dysfunction by inducing oxidative stress and inflammation [13]. Although *in vitro* evidence supports the PQ toxicity to neuronal cells, its *in vivo* effects (especially the chronic exposure) remain ambiguous because of the di-cationic nature of PQ in the plasma, which raises questions about its ability to cross the BBB.

Few studies focused on the signaling pathways involved in the neuronal toxicity after PQ poisoning. One of the suggested signaling pathways in the lung toxicity of PQ is PI3K/AKT pathway [14,15]. The PI3K/AKT pathway has been widely implicated in inflammation, cell growth, and survival [16]. However, the aberrant activation of PI3K/AKT pathway can lead to oxidative stress and cell death in immune cells [17]. In this study, we investigated the effect of chronic PQ exposure on the BBB damage and the underlying mechanisms in the rat and human brain microvascular endothelial cell models. Our study demonstrated that chronic PQ exposure could impair the BBB function and induce brain tissue damages. PI3K/AKT signaling pathway over-activation, the consequent IL-6 production, and increased oxidative stress may account for the inflammatory damages caused by PQ exposure, suggesting that targeting PI3K signaling could serve as a protective approach for PQ-induced brain damages.

## 2 Materials and methods

### 2.1 Animal treatments

A total of 30 adult male Sprague Dawley rats (weight about 260 g) were purchased from Cyagen Biosciences (Guangzhou, China) and housed in a pathogen-free animal house at 22°C with a 12 h light–dark cycle. Mice were randomly divided into three groups: control group were injected with normal saline; PQ treatment group was intraperitoneally injected with PQ (30 mg/kg) twice per week for 3 weeks. The intervention group (PQ + PI3K inhibitor) was intraperitoneally injected with PQ (30 mg/kg) and LY294002 (5 mg/kg) twice per week for 3 weeks. Then, the rats were sacrificed at the end of week 3 by cervical dislocation, and the brain tissues were dissected and stored at −80°C.

### 2.2 Cell treatments

Primary human brain microvascular endothelial (PHBME) cells were purchased from Procell (Wuhan, China). Cells were seeded into the six-well culture plates coated with 15 µg/mL rat tail collagen type I (Corning, NY, USA), and maintained in Microvascular Endothelial Cell Growth Medium-2 (Lonza, CA, USA) with 5% FBS and 100 U/mL of penicillin and 100 μg/mL of streptomycin (Beyotime, Beijing, China) in a 5% CO_2_ incubator at 37°C. Cells were treated with indicated concentration of PQ and LY294002 (50 μM) for 48 h.

### 2.3 Western blotting

RIPA buffer containing protease inhibitor cocktail (Beyotime, Beijing, China) was used to perform protein extraction. The cells were lysed on ice for 15 min and then centrifuged at 10,000×*g* at 4°C for 10 min. The protein concentration was determined by a BCA kit (Zeye Biotech, Shanghai, China). About 10 μg of protein was subjected to SDS-PAGE analysis and then transferred to a PVDF membrane. The membrane was incubated in 5% non-fat milk at 25°C for blocking, and then probed with primary antibodies overnight at 4°C. After washing, the membrane was further incubated with the secondary antibodies for 1 h at 25°C. The protein bands were developed using an enhanced chemiluminescence kit (Beyotime, Beijing, China), and the densitometry analysis was performed with Image J software (Bethesda, MD, USA). Primary antibodies used were PI3K (1/1,000, ab227204), p-PI3K (1/1,000, ab182651), Akt (1/2,000, ab8805), p-Akt (1/1,000, ab38449), and actin (1/1,000, ab8226); and secondary antibodies used were HRP-conjugated anti-rabbit IgG (1/5,000, ab6721) and anti-mouse IgG (1/5,000, ab97023).

### 2.4 Quantitative PCR (qPCR)

Trizol reagent (Invitrogen, Shanghai, China) was used to extract RNA from tissues and cells according to the instructions. The extracted total RNA was dissolved in diethylpyrocarbonate water and its concentration was measured with NanoDrop. Total RNA of 1 μg was used for reverse-transcription into cDNA using RevertAid First Strand cDNA Synthesis Kit (Thermo Fisher Scientific, CA, USA). The resulted cDNA was analyzed in a 7500 Real Time PCR System (Applied Biosystems, CA, USA) with the SYBR premix EX TAQ II kit (Takara, Dalian, China). The PCR cycling condition used was 95°C 2 min, 40 cycles of 95°C 30 s, 60°C 30 s and 72°C 60 s. The 2^–∆∆Ct^ method was used to analyze the relative expression level and GAPDH was used as the internal reference gene. All primer sequences were synthesized and purchased from Sangon Biotechnology Co., Ltd (Shanghai, China).

### 2.5 Evans blue dye penetration analysis

Evans blue reagent (Beyotime, Beijing, China) was administered to rats at a dose of 80 mg/kg. After 4 h of Evans blue injection, rats were transcardially perfused with autoclaved PBS. Then, brains were removed quickly and dissected into halves. One half of the tissue was dried in a drying oven at 150°C, on foil, for 48 h. After weighing the tissues, the tissue was placed in 200 µL of formamide in a microfuge tube for 48 h to extract the Evans blue. Then 50 µL of Evans blue-infused formamide was loaded into one well of a 96-well polystyrene plate, and the absorbance was recorded at OD620 for each sample using a microplate reader. The OD620 value was normalized to the dry tissue weight.

### 2.6 Histopathological studies

Hematoxylin and eosin (H&E) staining was used to study histological changes in the brain tissues. The brain tissues were fixed and embedded in paraffin wax. After cutting into 5 μm sections, the samples were staining using a H&E staining kit (Beyotime, Beijing, China) according to the manufacturer’s instruction. The section was rinsed using absolute ethanol three times and then mounted to a slide, and the images were collected under an inverse microscope.

### 2.7 Enzyme-linked immunosorbent assay (ELISA)

The inflammatory cytokines including IL-23, IL-6, IL-1β, IFNγ, and TNF-α were detected using corresponding ELISA kits (Jingkeye Biotechnology Co., Ltd, Shanghai, China) based on the supplier’s instructions. Results were calculated by measuring absorbance at 450 nm with microplate reader. Each sample was measured in triplicate, and the concentration of each cytokine was derived using the linear regression of the standards.

### 2.8 Cell scratch assay

Cells were seeded in a six-well plate to reach 80% confluence. A scratch wound was created using a sterile 200 μL pipette tip in the central region of each well. The floating cells were removed by changing the medium. The remaining cells were then incubated at 37°C for 48 h. Cell images were captured using an inverted light microscope (Leica AM6000 microscope) at 0 and 24 h.

### 2.9 Cell counting kit-8 (CCK-8)

PHBME cells were inoculated into a 96-well plate at a density of 3,000 cell/well, and cultured in a humidified cell culture incubator for 0, 24, 48, and 72 h. CCK8 reaction solution of 10 μL (Solarbio, Beijing, China) was added to each well at indicated time point, and the cells were incubated for 3 h in the incubator. The light absorption value in each condition was recorded at 530 nm wavelength on a Synergy H1 microplate reader (Winooski, Vermont, USA).

### 2.10 Lactate dehydrogenase (LDH) assay

Cytotoxicity was analyzed using Lactate Dehydrogenase Assay kit (Beyotime, Beijing, China). Briefly, the standard of LDH and the supernatant from cell culture were mixed with LDH assay buffer and LDH substrate. Standard wells = 50 µL standard dilutions; sample wells = 50 µL samples (adjust volume to 50 µL/well with LDH assay buffer). About 50 µL of reaction mix was added into each well for 30 min incubation. After gentle mix, the output was measured immediately at OD 450 nm using a microplate reader at 37°C.

### 2.11 ROS measurement

ROS levels were measured using the dichlorodihydrofluorescein diacetate (DCFDA) Cellular ROS Detection Assay Kit (ab113851; Abcam) according to the manufacturer’s instructions. The protein levels of cell or tissue lysates were quantified by a BCA kit (Zeye Biotech, Shanghai, China). About 100 μL of sample was stained with 20 μM of DCFDA solution for 30 min at 37°C in the dark. The resulting fluorescence intensity of each sample was measured using a microplate reader with maximum excitation and emission spectra of 495 and 529 nm, respectively. Fluorescence readings from unstained control wells were subtracted from reading values to account for background fluorescence. Values were normalized to protein content to determine the relative ROS levels between different treatment conditions.

### 2.12 Statistical analysis

The results were expressed as mean ± SD. Differences between the groups were determined by student’s *t*-test or one-way analysis of variance (ANOVA). Data at multiple time points were analyzed via two-way ANOVA. *p* < 0.05 was considered statistically significant.


**Ethical approval:** All animal experiments in our institute are approved by the Animal Ethics Committee of Kunming Medical University.

## 3 Results

### 3.1 PQ induces IL-6-associated inflammatory damages in the BBB and brain tissues

**Figure 1:**
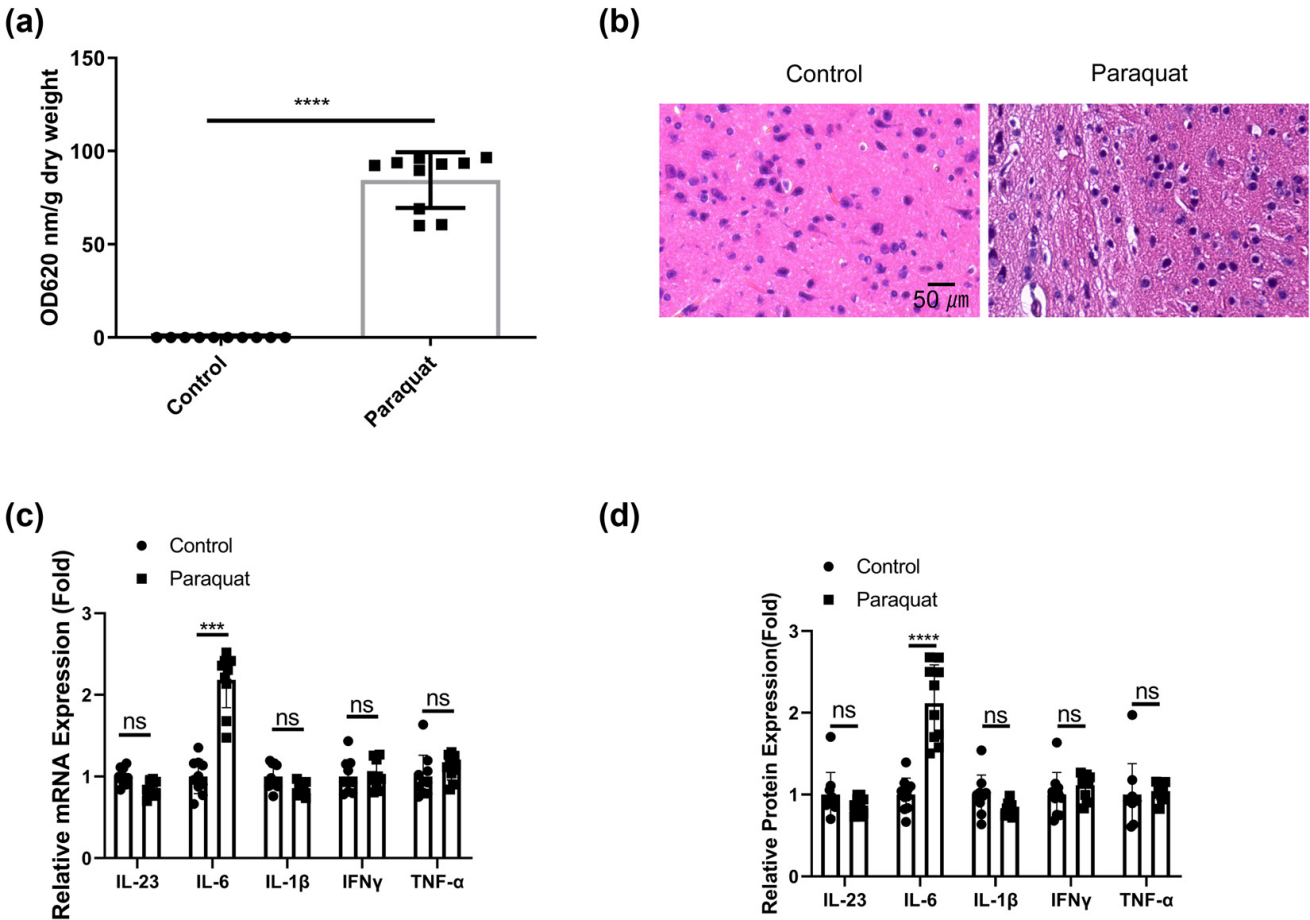
PQ induces IL-6-associated inflammatory damages in the BBB and brain tissues. Rat were administrated with normal saline or PQ for 3 weeks. (a). Evans blue dye penetration analysis and quantification in each group. (b) H&E staining of the brain tissues. (c) qRT-PCR and (d) ELISA analysis of inflammatory cytokines associated with neuroinflammation (IL-23, IL-6, IL-1β, IFNγ, and TNF-α) in the blood samples. *p < 0.05; **p < 0.01; ***p < 0.001; ****p < 0.0001.

To investigate the effect of chronic PQ exposure on the BBB and brain tissues, rats were administrated with PQ for 3 weeks. The rats were then administered with Evans blue reagent to examine the permeability of BBB. The quantitative analysis demonstrated a significant increase of Evans blue dye in the brain tissues after PQ treatment (Figure 1a), suggesting an impaired function of BBB. Accordingly, there was a higher degree of tissue damages in the brain tissues (Figure 1b). The levels of inflammatory cytokines associated with neuroinflammation including IL-23, IL-6, IL-1β, IFNγ, and TNF-α were examined in the blood samples. We observed that only IL-6 showed an significant increase after PQ exposure (Figure 1c). These data indicate that PQ induces IL-6-associated inflammatory damages in the BBB and brain tissues.

### 3.2 PQ impairs the function of PHBME cells at sub-toxic concentration

**Figure 2:**
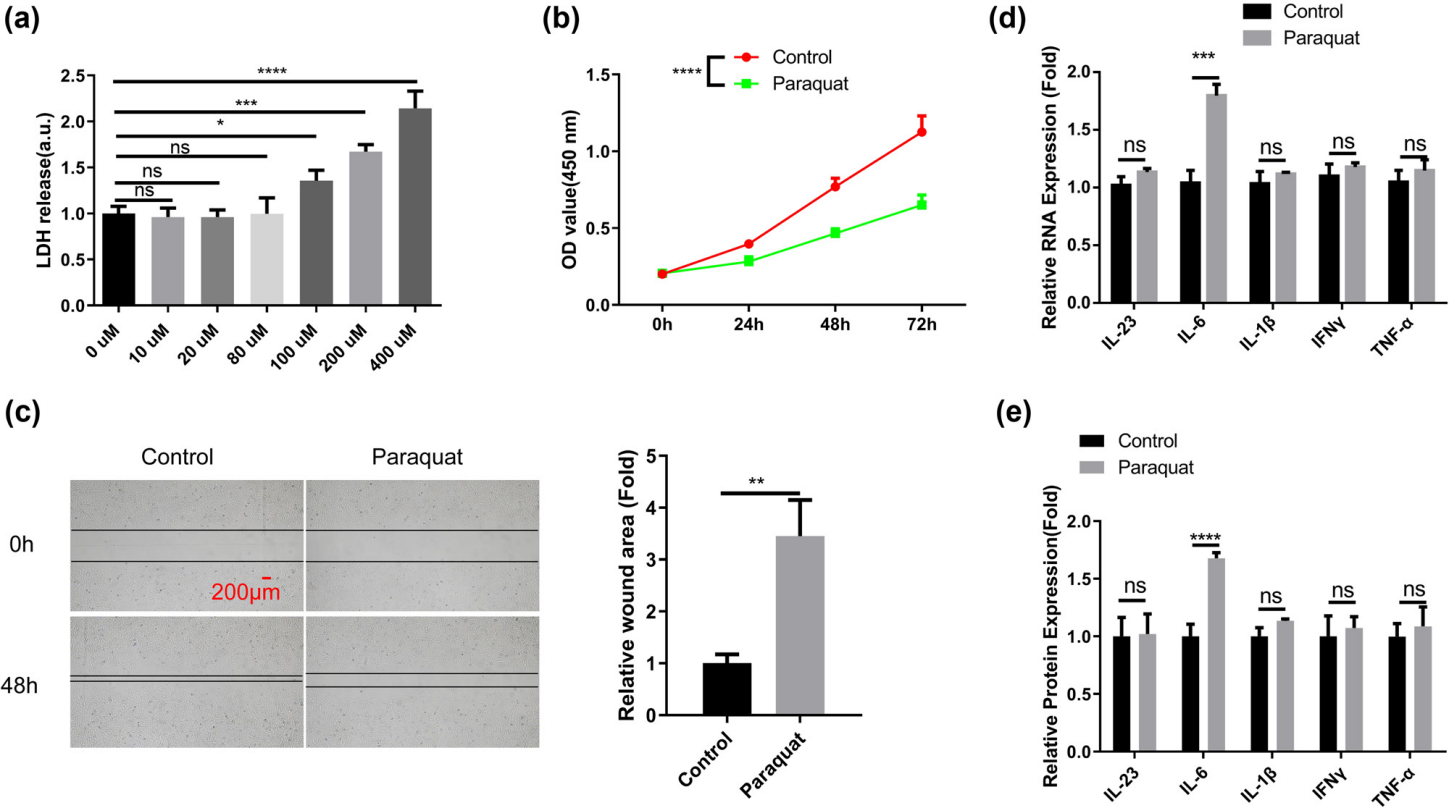
PQ impairs the function of PHBME cells at sub-toxic concentration. Brain endothelial vascular cells (PHBME cells) were treated with PQ for indicated duration. (a) LDH cytotoxicity assay after different doses of PQ treatment for 48 h. (b) CCK-8 cell proliferation of PHBME cells after sub-toxic PQ treatment (80 μM) for different durations. (c) Scratch assay in PHBME cells after sub-toxic PQ treatment (80 μM) for 48 h. (d) qRT-PCR and (e) ELISA analysis of inflammatory cytokines associated with neuroinflammation (IL-23, IL-6, IL-1β, IFNγ, and TNF-α) in PHBME cells after sub-toxic PQ treatment (80 μM) for 48 h. *p < 0.05; **p < 0.01; ***p < 0.001; ****p < 0.0001.

Next, we investigated the effect of PQ on PHBME cells, the *in vitro* model of brain endothelial vascular cells. LDH cytotoxicity assay revealed that PQ above 80 μM exerted significant toxic effect on PHBME cells (Figure 2a). We then utilized 80 μM as the sub-toxic dose to treat PHBME cells. PQ exposure could impair the cell proliferation and cell migration abilities (Figure 2b and c). In consistent with the *in vivo* data, PQ treatment significantly increased the mRNA and protein expression of IL-6 in PHBME cells (Figure 2d and e). These data imply that at sub-toxic dose PQ could impair the function of PHBME cells and promote the production of IL-6.

### 3.3 PQ activates PI3K/AKT signaling pathway

**Figure 3:**
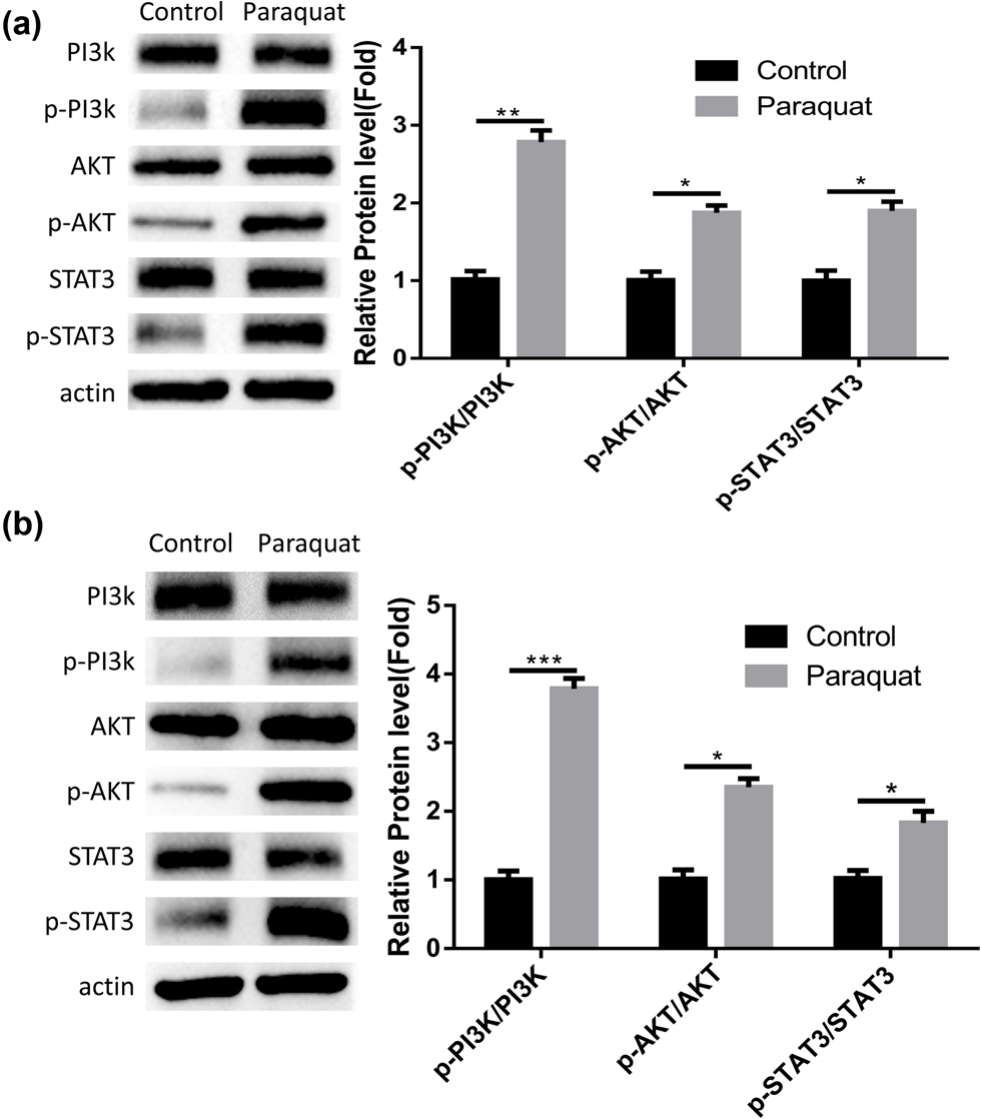
PQ activates PI3K/AKT signaling pathway. Western blot analyses of total PI3K, AKT, and STAT3, as well as the phosphorylation levels in the control group and PQ-treated group. (a) Data in PHBME cells. (b) Data in brain tissues of rat model. *p < 0.05; **p < 0.01; ***p < 0.001; ****p < 0.0001.

Since PQ was reported to over-activate PI3K/AKT signaling pathway in lung tissues [14,15], we performed western blot to check the status of PI3K/AKT signaling. In both the PQ-induced PHBME cells and the brain tissues of PQ-exposed rats, the phosphorylation level of PI3K and AKT kinases were significantly elevated, indicating that PQ exposure also activates PI3K/AKT signaling in brain tissues and the brain endothelial vascular cells (Figure 3a and b). Besides, we also examined the activation of STAT3, which is the downstream signaling transducer of IL-6 signaling. Consistent with the increased IL-6 production, the phosphorylation of STAT3 was significantly increased after PQ exposure (Figure 3a and b). Therefore, PI3K/AKT signaling over-activation may underlie the PQ-induced damages and IL-6 production.

### 3.4 PI3K inhibition attenuates the effect of PQ on PHBME cells

**Figure 4:**
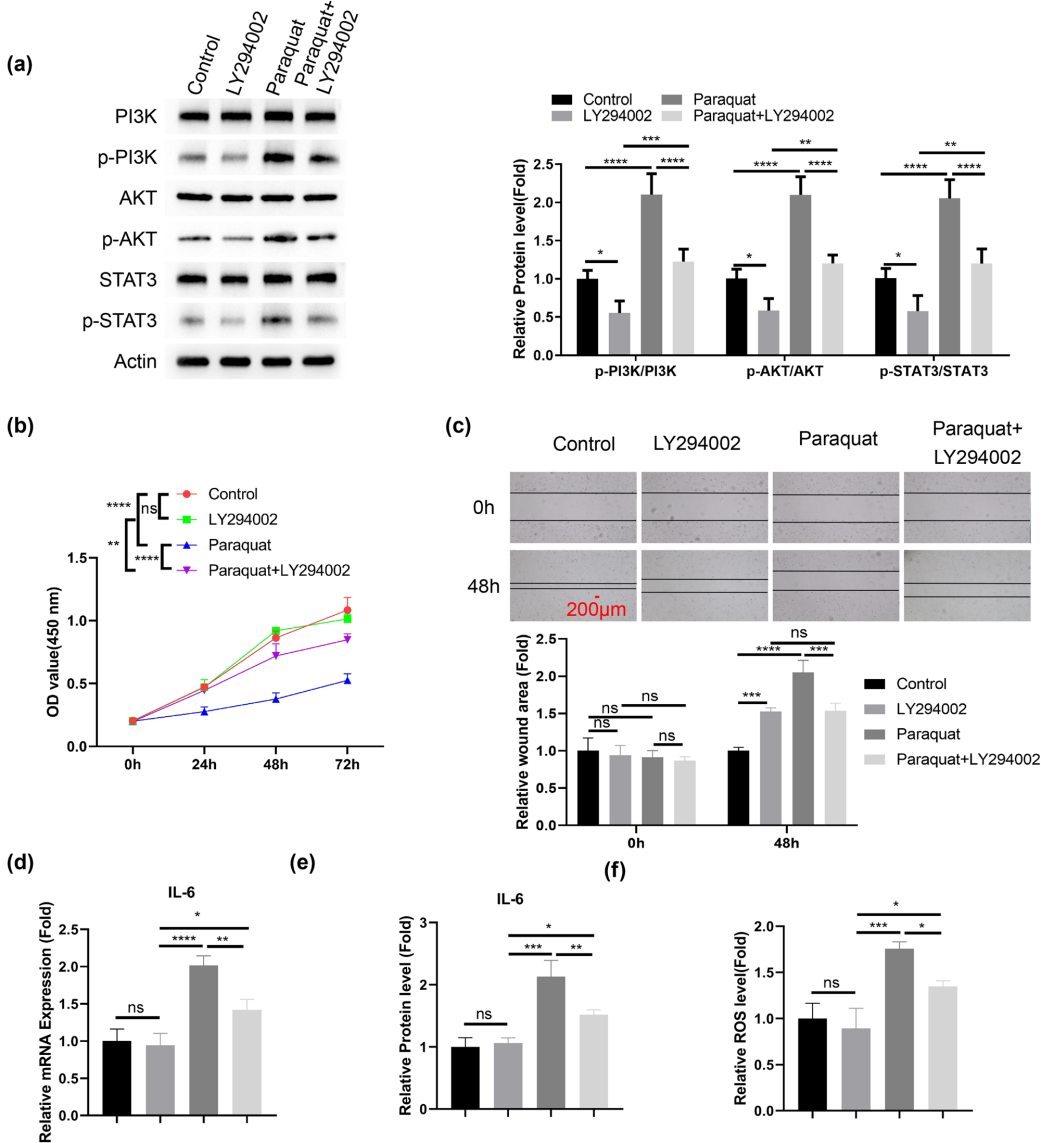
PI3K inhibition attenuates the effect of PQ on PHBME cells. PHBME cells were treated with sub-toxic dose of PQ in the presence or absence of PIK3 inhibitor (LY294002, 50 μM), or treated with PIK3 inhibitor alone. (a) Western blot analyses of total PI3K, AKT, and STAT3, as well as the phosphorylation levels. (b) CCK-8 cell proliferation. (c) Scratch assay. (d) qRT-PCR and (e) ELISA analysis of the IL-6 expression. (f) Detection of ROS levels. *p < 0.05; **p < 0.01; ***p < 0.001; ****p < 0.0001.

To confirm the contribution of PI3K/AKT signaling to PQ-induced effects, PHBME cells were treated with sub-toxic dose of PQ in the presence or absence of PIK3 inhibitor (LY294002). PI3K inhibition significantly suppressed the phosphorylation of PI3K/AKT after PQ induction, as well as the phosphorylation of STAT3 (Figure 4a). PI3K inhibition partially rescued the suppressed cell proliferation of PHBME cells upon PQ treatment (Figure 4b). PI3K inhibition also partially improved the migration of PHBME cells upon PQ treatment (Figure 4c). Meanwhile, PQ-induced IL-6 production was attenuated after PI3K inhibition (Figure 4d and e). We further observed that PQ-induced oxidative stress was attenuated by PI3K inhibitor (Figure 4f). Thus, these data suggest that PI3K inhibition mitigates PQ-induced damages in PHBME cells.

### 3.5 PI3K inhibition ameliorates PQ-induced inflammatory damages in the BBB and brain tissues

**Figure 5:**
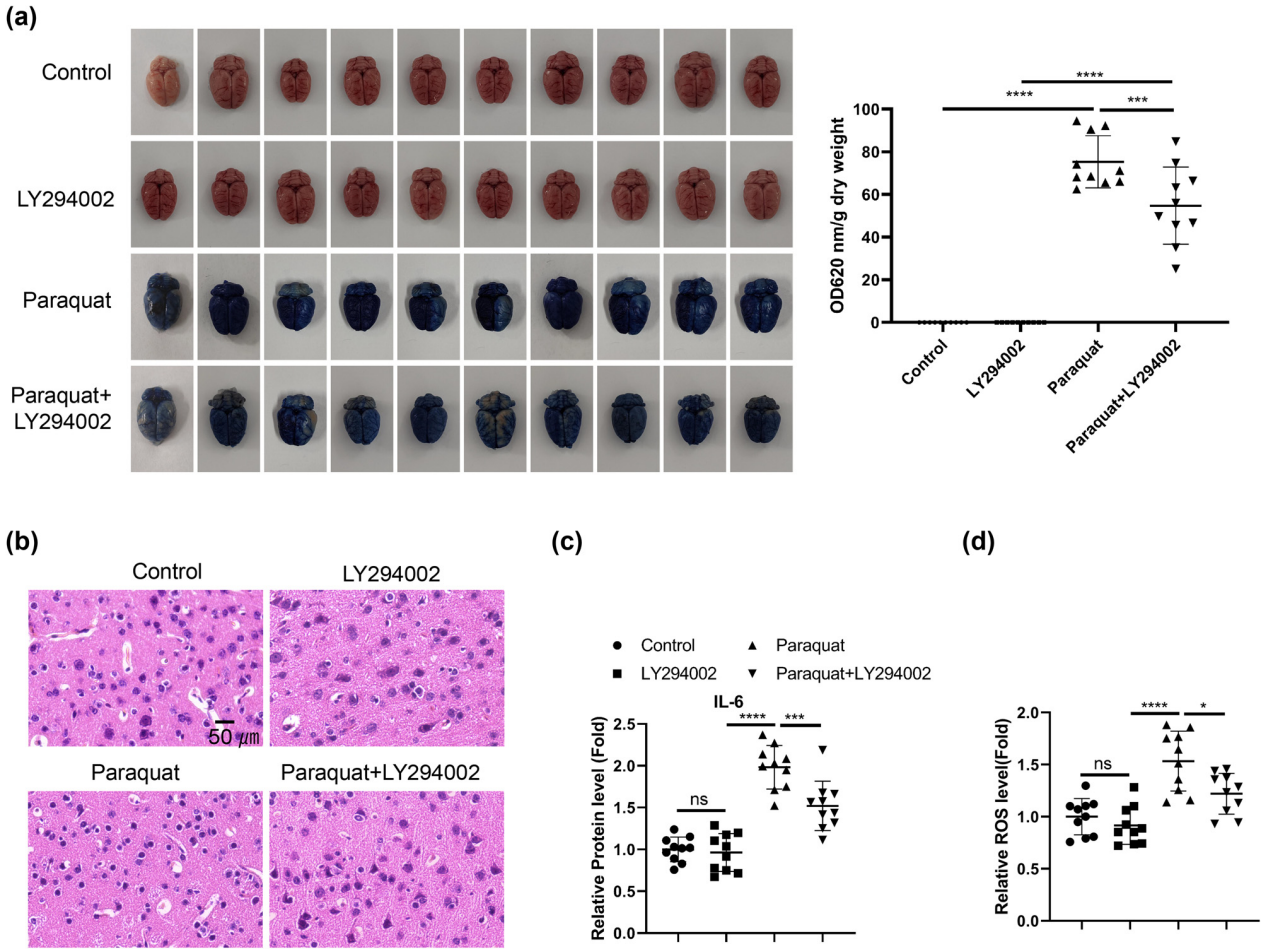
PI3K inhibition ameliorates PQ-induced inflammatory damages in the BBB and brain tissues. Rats were administrated with PQ for 3 weeks in the presence or absence of PI3K inhibitor or treated with PIK3 inhibitor alone. (a) Evans blue dye penetration analysis and quantification in each group. (b) H&E staining of the brain tissues. (c) ELISA analysis of inflammatory cytokine IL-6 in the blood samples. (d) Detection of ROS levels in the brain tissues. *p < 0.05; **p < 0.01; ***p < 0.001; ****p < 0.0001.

Lastly, we examined the potential effect of PI3K inhibition on PQ-induced brain damages. Rats were administrated with PQ for 3 weeks in the presence or absence of PI3K inhibitor. Evans blue dye penetration analysis showed that the application of PI3K inhibitor reduced the accumulation of Evans blue dye in the brain tissues after PQ exposure, which indicates the protective effect of PI3K inhibition on PQ-induced BBB damages (Figure 5a). Accordingly, the brain tissue damages induced by PQ were also ameliorated by PI3K inhibitor (Figure 5b), which was accompanied by the reduced levels of plasma IL-6 (Figure 5c). Meanwhile, PQ-induced ROS production in brain tissues was curtailed by PI3K inhibitor (Figure 5d). Together, these data suggest that PI3K inhibition could mitigate PQ-induced inflammatory damages in the BBB and brain tissues.

## 4 Discussion

Pesticides are broadly utilized in agriculture, including insecticides, herbicides, fungicides, and rodenticides [18]. PQ is a frequently used herbicide with neurotoxic effects after acute or chronic exposures, which incurs oxidative damages and inflammation to induce neurodegeneration [19]. PQ or the derivatives can possibly cross the BBB, and is mainly enriched in five different parts of the brain, including the cerebral ventricles, pineal gland, anterior portion of the olfactory bulb, hypothalamus, and area postrema [20]. Previous studies have shown that administration of PQ in mice can trigger an increase in pro-inflammatory cytokine levels in certain areas of the brain [21]. PQ has also been reported to cause oxidative stress in the dorsal striatum of male mice [22]. Nevertheless, the signaling pathway involved in PQ-induced neuroinflammation remains unclear. In our study, we demonstrated the chronic PQ exposure-induced damages in the BBB and brain tissues of rats. Besides, this effect was associated with an increased production of IL-6. IL-6 is a prominent pro-inflammatory cytokine involved in neuroinflammation. IL-6 mediated the activation of immune responses in Th17 cells and Th1 cells in experimental autoimmune encephalomyelitis [23]. Besides, recent evidence suggests the detrimental effect of IL-6-mediated inflammation on the integrity of the BBB. For example, IL-6 production triggered by periodontal inflammation induces neuroinflammation and disrupts the BBB function [24]. The neutralization of IL-6 could ameliorate neuromyelitis optica spectrum disorder and preserve the integrity of the BBB [25]. Together, our data and previous evidence pinpoint IL-6 as a detrimental inflammatory factor associated with the undermined function of the BBB in pathophysiological conditions.

Emerging evidence suggests that the deregulation of PI3K/AKT pathway is implicated in the sepsis-induced brain damages [26]. Recently, the deregulation of PI3K/AKT pathway has attracted increasing research attention for potential neuroprotection intervention in brain diseases [27,28]. Although previous studies has implicated the over-activation of PI3K/AKT in PQ-induced lung damages [14,15], whether this signaling pathway underlie PQ-induced brain damages is unclear. In our data, we showed that PQ also heavily activated the PI3K/AKT signaling pathway in both the cell and animal model. In addition, PI3K inhibitor not only rescued the cellular function of PHBME cells upon PQ exposure, but also protect against PQ-induced damages in the BBB and brain tissues. These data were consistent with the previous study in PQ-induced lung damages.

Notably, PI3K inhibition also attenuates the production of IL-6 after PQ treatment. A previous study reported that in Rheumatoid arthritis, IL-6 is a key pro-inflammatory cytokine induced by sphingosine-1-phosphate through the activation of PI3K, MEK/ERK, and NF-κB signaling cascades [29]. How PI3K/AKT signaling activates IL-6 production warrants further investigation. We could not exclude the possibility that other signaling pathways are also involved in PQ-induced IL-6 production. For instance, there is evidence that PI3K/AKT pathway also affects the protein synthesis system via regulating downstream mTOR/S6K/4EBP1 axis [30,31]. It is possible that PQ interferes with protein synthesis to exert cytotoxicity in the BBB. Therefore, additional work is required to comprehensively characterize PQ-induced signaling processes in the BBB.

There is evidence that IL-6 receptor is expressed on the surface of human vascular endothelial cells, and that the expression is positively regulated by pro-inflammatory stimuli [32]. Thus, the over-production of IL-6 may exert a positive-feedback effect to amplify the IL-6-mediated signaling cascade by upregulating its own receptor. Besides, IL-6 trans-signaling induces the release of the chemokine Monocyte Chemoattractant Protein-1 in human vascular endothelial cells [32]. This may in turn serve as a pro-inflammatory signal to recruit monocytes to vascular endothelial cells, exacerbating the inflammatory damages. Furthermore, we also observed elevated ROS levels after PQ exposure, which is concomitant with IL-6 production. Neuroinflammation and oxidative stress have been recognized as major detrimental factors contributing to the BBB disruption [33,34]. PQ-induced oxidative stress has been widely reported in neurons, vascular endothelial cells and immune cells [35–37]. Therefore, IL-6-mediated inflammation and elevated oxidative stress may jointly drive pathogenic damages on the BBB after prolonged PQ exposure. Since nuclear factor erythroid 2-related factor 2 (NRF2) is a key mediator of antioxidant response [38], whether the dysregulation of NRF2 is involved in PQ-induced oxidative stress warrants further investigation.

One key limitation of this study is that we did not definitively establish a direct causal link between increased IL-6 levels and the BBB disruption caused by PQ exposure. While the data clearly showed that PQ increased IL-6 expression concomitantly with the BBB damage, the precise mechanisms by which IL-6 may impact barrier integrity are not elucidated. Further experiments are needed to determine if IL-6 directly impairs barrier function, or if it acts indirectly through inflammatory signaling cascades and recruitment of other cell types like immune cells. Targeted neutralization or knockdown of IL-6 could help clarify the necessity of this cytokine in mediating PQ-induced barrier disruption. Additionally, the study is limited by use of a single cell model (PHBME cells) which may not fully recapitulate the complex multicellular microenvironment of the BBB *in vivo*. Future studies using other barrier models like 3D microfluidic chips or stem cell-derived brain endothelial cells could provide complementary insights. Overall, while this work implicates IL-6 as a potential key mediator, more research is still required to conclusively determine its direct roles and mechanisms in the deleterious effects of PQ on the BBB.

## 5 Conclusions

Our study demonstrated that chronic PQ exposure can cause significant pathological disruption in the brain tissues and the BBB. IL-6 was significantly upregulated after PQ exposure in both the animal and cell model. The over-activation of PI3K/AKT and the consequent IL-6 production may account for the inflammatory damages caused by PQ exposure. Thus, our data suggest that apart from PI3K inhibition, IL6 blocking may also serve as an intervention strategy to ameliorate PQ-induced damages in the BBB and brain tissues.
